# Altered germline cyst formation and oogenesis in *Tex14* mutant mice

**DOI:** 10.1242/bio.058807

**Published:** 2021-06-22

**Authors:** Kanako Ikami, Nafisa Nuzhat, Haley Abbott, Ronald Pandoy, Lauren Haky, Allan C. Spradling, Heather Tanner, Lei Lei

**Affiliations:** 1 Buck Institute for Research on Aging, 94949, Novato, CA, USA; 2 Department of Cell and Developmental Biology, University of Michigan Medical School, 48109, Ann Arbor, MI, USA; 3 Department of Embryology, Carnegie Institution for Science, 21218, Baltimore, MD, USA

**Keywords:** Germline cysts, Oogenesis, Oocytes, TEX14

## Abstract

During oocyte differentiation in mouse fetal ovaries, sister germ cells are connected by intercellular bridges, forming germline cysts. Within the cyst, primary oocytes form via gaining cytoplasm and organelles from sister germ cells through germ cell connectivity. To uncover the role of intercellular bridges in oocyte differentiation, we analyzed mutant female mice lacking testis-expressed 14 (TEX14), a protein involved in intercellular bridge formation and stabilization. In *Tex14* homozygous mutant fetal ovaries, germ cells divide to form a reduced number of cysts in which germ cells remained connected via syncytia or fragmented cell membranes, rather than normal intercellular bridges. Compared with wild-type cysts, homozygous mutant cysts fragmented at a higher frequency and produced a greatly reduced number of primary oocytes with precocious cytoplasmic enrichment and enlarged volume. By contrast, *Tex14* heterozygous mutant germline cysts were less fragmented and generate primary oocytes at a reduced size. Moreover, enlarged primary oocytes in homozygous mutants were used more efficiently to sustain folliculogenesis than undersized heterozygous mutant primary oocytes. Our observations directly link the nature of fetal germline cysts to oocyte differentiation and development.

## Abstract (precis)

**Summary:** Altered germline cyst formation and fragmentation due to defective germ cell connectivity leads to changes in oocyte differentiation and development in *Tex14* mutant mice.

## INTRODUCTION

In mammals, primordial follicles, each contains a primary oocyte and a single layer of pregranulosa cells, serve as the only source for sustaining oogenesis and ovarian function in adult females (Baker, 1963; Richardson et al., 1987; Faddy and Gosden, 1996). Primordial follicles form in the fetal and neonatal mammalian ovaries, where pregranulosa cells enclose individual primary oocytes to assemble into the follicular structure. In mice, the process of oocyte differentiation (i.e. primary oocyte formation) begins when primordial germ cells (PGCs) proliferate with incomplete cytokinesis from embryonic day 10.5 (E10.5) to E14.5. Germ cells derived from the same PGC remain connected by intercellular bridges, forming germline cysts. As germ cells divide, germline cysts fragment into smaller cysts of various sizes. On average, each PGC produces five cysts, each containing six germ cells in E14.5 ovaries. Within the cyst, intercellular bridges enable germ cells to exchange signaling molecules, so that sister germ cells undergo synchronized mitosis and meiosis in the cyst (Pepling and Spradling, 1998; Lei and Spradling, 2013b). Moreover, intercellular bridges enable organelle exchange between sister germ cells. By electron microscopy (EM), mitochondria and endoplasmic reticulum (ER) were observed in the lumen of intercellular bridges joining two E14.5 germ cells (Pepling and Spradling, 2001). As oocyte differentiation progresses, intercellular bridges detach from the cell membrane, which enlarges the connectivity between sister germ cells around E17.5 and facilitates transport of large organelles, such as Golgi complexes. Cytoplasmic transport within cysts enables ∼20% of the E14.5 germ cells to differentiate into primary oocytes containing an enlarged cytoplasmic volume and organelles by postnatal day 4 (P4), while the remaining fetal germ cells undergo apoptosis after donating these materials (Lei and Spradling, 2016).

During mammalian gametogenesis, intercellular bridges are found in fetal ovaries, fetal testes, and adult testes (Ruby et al., 1969; Gondos, 1973; Ruby et al., 1970; DW, 1958; Greenbaum, 2006). Studies in adult mouse testes demonstrate that the testis-expressed 14 (TEX14) protein is essential for blocking cytokinesis and converting transient midbody rings into stable intercellular bridges (Greenbaum et al., 2007; Iwamori et al., 2010; Kim et al., 2015). During cytokinesis in somatic cells, centrosomal 55-kDa protein (CEP55) is recruited to the centralspindlin complex at the midbody region that contains mitotic kinesin-like protein 1 (MKLP1) and Rac GTPase-activating protein (RacGAP) (Mishima et al., 2002). Two other proteins, ALG-2 interacting protein X (ALIX) and tumor susceptibility gene 101 protein (TSG101) are further recruited to the midbody by interacting with CEP55. The Glycine (G)-proline (P)-proline (P)-X-X-X-tyrosine (Y) (GPPX3Y) motifs in ALIX and TSG101 interact with CEP55, a process that facilitates abscission between two cells undergoing cytokinesis (Morita et al., 2007; Iwamori et al., 2010). During germ cell division in adult testes, TEX14 protein is recruited to the midbody where the GPPX3Y motif of TEX14 binds strongly to CEP55 and blocks the interaction between CEP55, ALIX and TSG101 (Fig. S1). This process converts midbodies into stable intercellular bridges (Iwamori et al., 2010; Kim et al., 2015). Testes of *Tex14* mutant mice are smaller in size but contain normal spermatogonia stem cells. Stable intercellular bridges are absent in postnatal mutant testes, and the first wave of spermatogenesis halts before the completion of the first meiotic division. The defect in meiosis due to lack of intercellular bridges causes infertility in *Tex14* mutant male mice (Greenbaum et al., 2006). *Tex14* mutant females are fertile, but their ovaries contain fewer oocytes compared to wild-type females. Stable intercellular bridges are not observed in *Tex14* mutant neonatal ovaries, although germ cells are in clusters (Greenbaum et al., 2009). Due to technical difficulties, important questions, such as whether ovarian fetal germ cells still form germline cysts and remain connected without stable intercellular bridges, and whether cytoplasmic enrichment during oocyte differentiation takes place in *Tex14* mutant fetal ovaries, remain to be addressed.

In the present study, we report that *Tex14* homozygous mutant female germ cells form syncytial cysts or cysts with fragmented cell membrane without normal intercellular bridges, and produce a greatly reduced number of primary oocytes of increased size. However, the enlarged homozygous mutant primary oocytes are used efficiently for folliculogenesis, partially compensating for their reduced number. In contrast, in *Tex14* heterozygous mutant germ cells, reduced amounts of TEX14 protein leads to decreased cyst fragmentation and a slightly increased number of primary oocytes of reduced size. But the undersized heterozygous mutant primary oocytes turn over at an accelerated rate. These observations show that despite losing normal intercellular bridges, *Tex14* mutant females continue to produce oocytes using abnormal germline cysts, and suggest that the cytoplasmic content of the primary oocyte may strongly influences the efficiency of oocyte usage in adult ovaries.

## RESULTS

### Formation of germline cysts lacking stable intercellular bridges in *Tex14* homozygous mutant gonads

To directly uncover whether or not PGCs divide to form germline cysts in the absence of TEX14 protein, we first conducted single-cell lineage tracing in mutant mice. As shown in our previous study, a single low dose of tamoxifen injection at E10.5 induces the expression of reporter YFP only in one or two widely separated PGCs, without causing detectable defects in oocyte differentiation (oocyte number and oocyte volume) (Mork et al., 2012; Lei and Spradling, 2013b). The progeny germ cells generated from individual PGCs remain nearby without migrating to the other locations of the ovary during oocyte differentiation, thus a germ cell clone derived from a single PGC can be recognized unambiguously. This approach allows the progeny cells generated from individual PGCs to be followed, even when sister germ cells are not connected due to cyst fragmentation. Individual YFP-positive germ cell clusters in a germ cell clone were defined as cysts of a clone. The number of YFP-positive germ cells of each YFP-positive germ cell cluster was quantified as the size of each cysts.

**Fig. 1.**
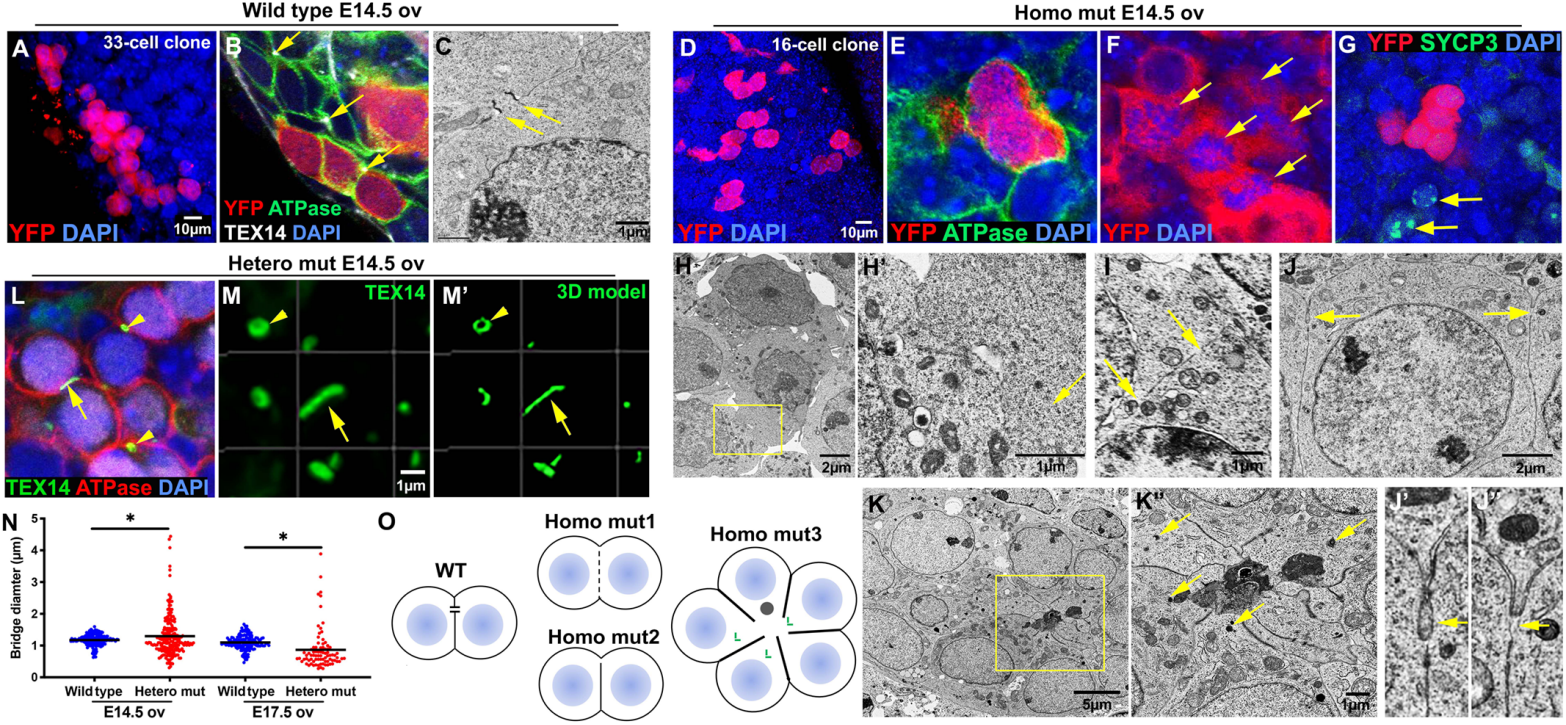
**Defects in intercellular bridges in *Tex14* mutant germline cysts.** (A) A YFP-positive germ cell clone containing germline cysts revealed by single-PGC lineage tracing in the E14.5 wild-type ovary (*n*=30 ovaries). (B) Immunostaining showing wild-type cyst germ cells were connected by intercellular bridges (arrows) located at the end of the cell–cell interface (*n*=6 experiments). (C) An electron microscopic image showing an intercellular bridge with cell membrane attached to both sides of the bridge (*n*=6 ovaries). (D) A YFP-positive germ cell clone containing germline cysts in the E14.5 homozygous mutant (homo mut) ovary (*n*=32 ovaries). (E) Immunostaining showing clustered homozygous mutant cyst germ cells with no detectable cell membrane and intercellular bridges between two cyst germ cells (*n*=6 ovaries; *n*=34 lineage-labeled germ cells). (F) Synchronized mitosis revealed by clustered, dividing germ cells in the homozygous mutant ovary (arrows) (*n*=6 ovaries). (G) Cyst germ cells were synchronized in entering meiosis. Among YFP-positive cyst germ cells, none were in meiosis. Two nearby germ cells (arrows) were in meiotic division (*n*=6 ovaries). (H–K″) Germ cell connectivity defects observed in E14.5 homozygous mutant fetal ovaries. Clustered germ cells were found frequently in the E14.5 homozygous mutant fetal ovary with fragmented cell membrane that leaves opening (arrow) between two germ cells (H–I′). (J–J″) Three homozygous mutant germ cells were connected with a narrow opening (arrows) at the end of the shared cell membrane. (K–K″) Homozygous mutant germ cells formed a rosette structure where cell–cell membrane form junctions and centrosomes (arrows) were near the open area in the center of the rosette (*n*=6 ovaries). (L) A fragmented TEX14 positive intercellular bridge (arrow) and two circular intercellular bridges (arrow heads) observed in E14.5 heterozygous mutant (hetero mut) ovaries (*n*=6 ovaries). (M–M′) A fragmented TEX14 positive intercellular bridge (arrow) and a circular intercellular bridge (arrow head) viewed in 3D. (N) Measurements of TEX14 positive bridge diameters in E14.5 and E17.5 heterozygous mutant ovaries (*n*=6 ovaries). Unpaired *t*-test was used to compare the size between wild-type and heterozygous mutant bridges. *P* value=0.0133 (E14.5), *P* value=0.0001(E17.5). (O) Diagram of germ cell connectivity observed in E14.5 wild-type (WT) and homozygous mutant (homo mut) ovaries.

**Fig. 2.**
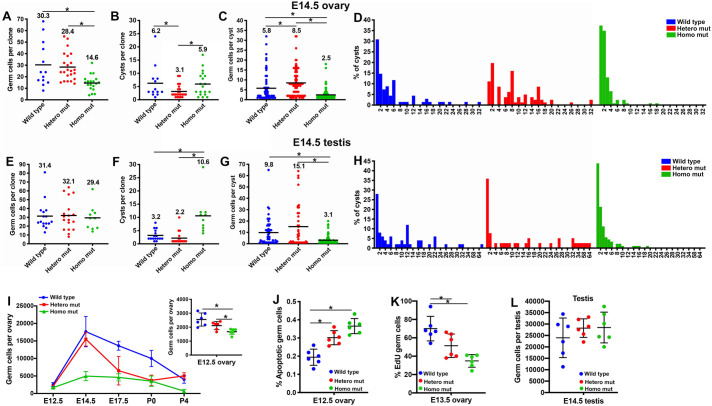
**Altered germline cyst formation and fragmentation in *Tex14* mutants.** Numbers of germ cells in each lineage-labeled clone in E14.5 ovaries (A) and testes (E). Numbers of cysts in each clone in E14.5 ovaries (B) and testes (F). Numbers of germ cells in each cyst in E14.5 ovaries (C) and testes (G). The distribution of cysts by size in E14.5 ovaries (D) and E14.5 testes (H). These analyses were based on *n*=30 wild-type, *n*=36 hetero mutant and *n*=32 homo mutant ovaries, and *n*=22 wild-type, *n*=42 hetero mutant and *n*=20 homo mutant testes. The bar and number on the top represent the mean value. (I) Changes in germ cell number during oocyte differentiation (*n*=6 ovaries). (J) Percentage of cleaved-PARP-positive germ cells in the E12.5 ovary (*n*=6 ovaries). (K) Percentage of EdU-positive mitotic germ cells in the E13.5 ovary (*n*=6 ovaries). (L) Number of germ cells in E14.5 testes (*n*=6 testes). Error bars present standard deviations in I–L. One-way ANOVA was used for statistical analyses, * indicates significant difference with *P* value <0.05.

**Fig. 3.**
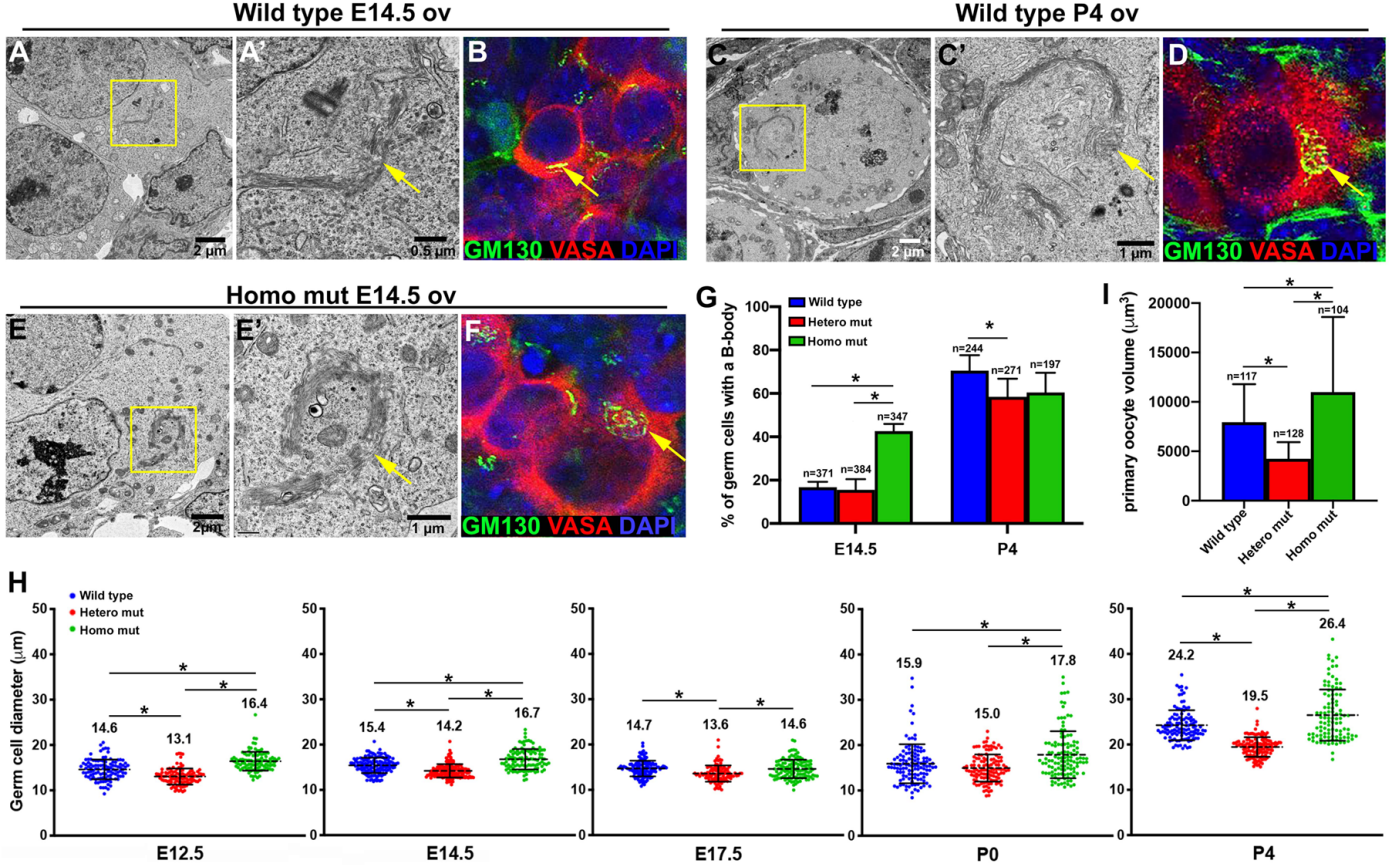
**Precocious cytoplasmic enrichment during oocyte differentiation in *Tex14* homozygous mutant ovaries.** (A,B). A morphologically lineal Golgi complex (arrow) was observed in the germ cells of E14.5 wild-type ovaries by EM (A, A′, *n*=6 ovaries, *n*=247 germ cells) and immunostaining (B, *n*=6 ovaries, *n*=312 germ cells). (C,D) In the primary oocytes of P4 wild-type ovaries, a spherical structure of Golgi complexes (B-body, arrow) was observed by EM (C,C′, *n*=6 ovaries, *n*=114 germ cells) and immunostaining (D, *n*=6 ovaries, *n*=317 germ cells). (E,F) In the germ cells of E14.5 homozygous mutant ovaries, the B-body (arrow) was observed by EM (E,E′, *n*=6 ovaries, *n*=87 germ cells) and immunostaining (F, *n*=6 ovaries, *n*=132 germ cells). (G) Percentage of germ cells containing a B-body in wild-type and *Tex14* mutant ovaries (*n*=6 ovaries). (H) Changes in germ cell diameter during oocyte differentiation in wild-type and *Tex14* mutant ovaries (*n*=6 ovaries). (I) Average volumes of primary oocytes in wild-type and *Tex14* mutant P4 ovaries (*n*=6 ovaries). Error bars present standard deviations in G–I. One-way ANOVA was used for statistical analyses, * indicates significant difference with *P* value <0.05.

In the present study, after a single low dose of tamoxifen injection at E10.5, fetal ovaries and testes were collected at E14.5 when mitotic germ cell division has been completed, and stained with a GFP antibody to identify the lineage-labeled germ cells in the gonad. In wild-type ovaries, clustered YFP-positive cyst germ cells were readily observed (Fig. 1A). These germ cells each had an intact cell membrane (stained by an antibody to Na^+^K^+^-ATPase) and were connected by TEX14-positive intercellular bridges (Fig. 1B). By EM, these intercellular bridges can be morphologically recognized joining adjacent germ cells (Fig. 1C,O). Lineage-labeled clustered cyst germ cells were found in *Tex14* homozygous mutant ovaries at a similar frequency as in wild-type gonads (Fig. 1D; Fig. S2). The connectivity of lineage-labeled homozygous mutant germ cells was shown by gaps in the cell membranes (labeled by the antibody to Na^+^K^+^-ATPase) between sister germ cells (Fig. 1E), and by the fact they retained synchronized mitosis and meiosis (Fig. 1F,G; Fig. S3). To further characterize the germ cell connectivity between mutant cyst germ cells, we examined germ cell morphology in E14.5 ovaries by EM (Fig. 1H–K). Germ cell clusters without interspersed somatic cells were observed frequently in homozygous mutant ovaries. Typical intercellular bridges observed in wild-type ovaries were not found in homozygous mutant ovaries. Of the clustered germ cells (*n*=130) that were found to be connected in EM images, three means of connectivity were observed: (1) 36.2% of these germ cells were connected due to fragmented membrane. Organelles were observed between the fragmented membranes (Fig. 1H–I,O); (2) 6.15% of these germ cells were connected via a small membrane gap between two adjacent germ cells with intact cell membrane, which may result from the failure of forming/stabilizing intercellular bridges (Fig. 1J–J″,O); (3) 10.8% of these germ cells formed rosette syncytia, where germ cell membranes merge at the center of the syncytium. Germ cell centrosomes were often located at the center of such syncytia where the cell membranes merge (Fig. 1K–K′,O). The remainder showed intact cell membrane. TEX14 was needed for intercellular bridge formation in fetal testes as well. Clonally labeled, clustered germ cells were also found in E14.5 homozygous mutant fetal testes (Fig. S4). However, intercellular bridges were not observed by EM.

TEX14-positive bridges were still present in *Tex14* heterozygous mutant ovaries (Fig. 1L, arrowheads). However, compared with bridges in wild-type ovaries, heterozygotes ovaries contained a higher frequency of fragmented bridges (Fig. 1L–M′, arrow; Fig. S5). To estimate the difference in bridge size between wild-type and heterozygous mutant ovaries, we measured the diameter/largest cross section of the TEX14 positive bridges in E14.5 and E17.5 ovaries. Bridges in E14.5 wild-type ovaries measured 1.2 µm on average, ranging from 0.6 to 1.6 µm. However, bridges in E14.5 heterozygous mutant ovaries showed a much wider size spectrum, with an average of 1.3 µm while ranging from 0.3 to 4.4 µm (Fig. 1N). Increased size dispersion was observed in E17.5 ovaries as well; average bridge diameter was 1.1 µm in wild-type versus 0.87 µm in heterozygous mutant ovaries (Fig. 1N). Bridge stability may be decreased under these conditions due to reduced amount of TEX14 protein in heterozygous mutant ovaries (Greenbaum et al., 2007) (Fig. S6). These observations show that TEX14 is haploinsufficient for normal intercellular bridge production in ovaries. The fact that both enlarged and condensed bridges are observed in heterozygous mutant ovaries suggests that the tension at the midbody ring may be dynamic and involve both expansion and contraction.

### *Tex14* heterozygous and homozygous mutant germ cells showed contrasting pattens of germline cyst formation and fragmentation

To compare the properties of germline cysts formed with normal intercellular bridges in wild-type gonads, of cysts with unstable intercellular bridges in heterozygous mutant gonads, and of cysts lacking intercellular bridges in homozygous mutant gonads, we analyzed: (1) the number of germ cells in each clone; (2) the number of cysts in each clone and (3) the number of germ cells in each cyst in E14.5 ovaries and testes.

On average, each PGC produced clones of 30.3±19.4 cells in wild-type, 28.4±12.0 cells in heterozygous-mutant and 14.6±6.8 cells in homozygous-mutant ovaries at E14.5 when germ cell mitosis ceased (Fig. 2A). Compared with wild-type cysts, heterozygous mutant cysts fragmented less and thus produced larger cysts (Fig. 2B,C). When cysts were profiled by size, we found that heterozygous mutant cysts had a higher chance of containing two cells, four cells, eight cells and 16 cells, i.e. cysts tended to remain connected, and thus to undergo synchronized mitosis to produce cysts at the size of the power of 2. This observation is consistent with the result that heterozygous mutant cysts fragmented less frequently. Homozygous mutant germ cell clones contained about the same number of cysts as wild type, but they were in a smaller size on average, with most of them containing approximately two to eight cells. The ratio of single germ cells was slightly increased in homozygous mutant E14.5 ovaries (30.9% in wild type versus 37.4% in homozygous mutant) (Fig. 2D)*.* These results indicate that an increased number of single cells are produced due to cyst fragmentation during germ cell mitosis in homozygous mutant ovaries.

Germ cell clones observed in E14.5 testes of the three genotypes were about the same size (Fig. 2E), but fragmented very differently. Similar to the cyst fragmentation patterns observed in fetal ovaries, heterozygous mutant cysts fragmented less than wild-type cysts and each cyst contained more germ cells (15.1±18.4 cells). Homozygous mutant cysts fragmented more extensively, thus a greater number of cysts of reduced size (3.1±3.6 cells) were observed compared with wild-type cysts (Fig. 2F,G). Notably, the proportion of single cells increased significantly in homozygous mutant male cysts (Fig. 2H).

To elucidate the change in germ cell numbers during the entire process of oocyte differentiation from E12.5 to P4, we quantified germ cell numbers in the fetal and neonatal ovary. In all three genotypes, fetal germ cell number per ovary reached a maximum at E14.5 and began to decline afterwards. On average 24.6%, 36.5%, and 16.2% of the E14.5 fetal germ cells differentiated into primary oocytes in wild-type, heterozygous and homozygous mutant ovaries respectively (Fig. S7). Although differences in the number of germ cells per ovary in three genotypes could first be detected at E12.5, they increased significantly by E14.5 (Fig. S8). An E14.5 homozygous mutant ovary only contained 28% as many germ cells as a wild-type ovary (Fig. 2I). The decreased germ cell number in E14.5 homozygous mutant ovaries is a combined result of increased germ cell apoptosis, measured as the percentage of cleaved-PARP-positive cells (Fig. 2J), and significantly reduced germ cell mitosis, measured as the percentage of EdU-positive germ cells (Fig. 2K). Interestingly, the defect in germ cell mitosis was observed in homozygous mutant fetal ovaries, but not in testes. Germ cell numbers were comparable in E14.5 testes of the three genotypes (Fig. 2L).

### Precocious cytoplasmic enrichment in *Tex14* homozygous mutant germline cysts

In wild-type ovaries, as cytoplasmic transport within the germline cyst progresses, future oocytes become enriched in organelles, Golgi complexes gradually organize into a spherical structure, forming the Balbiani body (B-body) in mouse primary oocytes (Lei and Spradling, 2016). In E14.5 wild-type germ cells, a linear-shaped Golgi complex was observed by EM and immunostaining of the Golgi-specific protein GM130 (Fig. 3A,B). As oocyte differentiation ceases in P4 ovaries, the B-body was found in 70.5±7.1% of wild-type primary oocytes (Fig. 3C,D,G). In contrast, clustered Golgi complexes (B-body) were already observed using EM and immunostaining by E14.5 homozygous in mutant germ cells (Fig. 3E,F). Compared with E14.5 wild-type ovaries, in which only 16.8±2.5% of the germ cells contained a B-body, 42.6±3.4% of E14.5 homozygous mutant germ cells contained a B-body, indicating that cytoplasmic enrichment takes place precociously in these cysts (Fig. 3G). This observation was further confirmed by the measurement of germ cell diameters during oocyte differentiation. Homozygous mutant germ cells were larger than wild type in mean diameter starting from E12.5; in contrast, the average diameter of heterozygous mutant cells was smaller than wild-type cells. These relative differences in germ cell size remained during oocyte differentiation (Fig. 3H). By P4, homozygous mutant primary oocytes were 1.4 times bigger than wild-type primary oocytes on average, and 60.5±9.1% of these primary oocytes contained a B-body. On average, heterozygous mutant primary oocytes were only 53% of the volume of wild-type primary oocytes, and a significantly reduced number of these primary oocytes (58.5±8.3%) contained a B-body compared with wild type (Fig. 3G,I).

### Dynamics of folliculogenesis in *Tex14* mutant adult ovaries

**Fig. 4.**
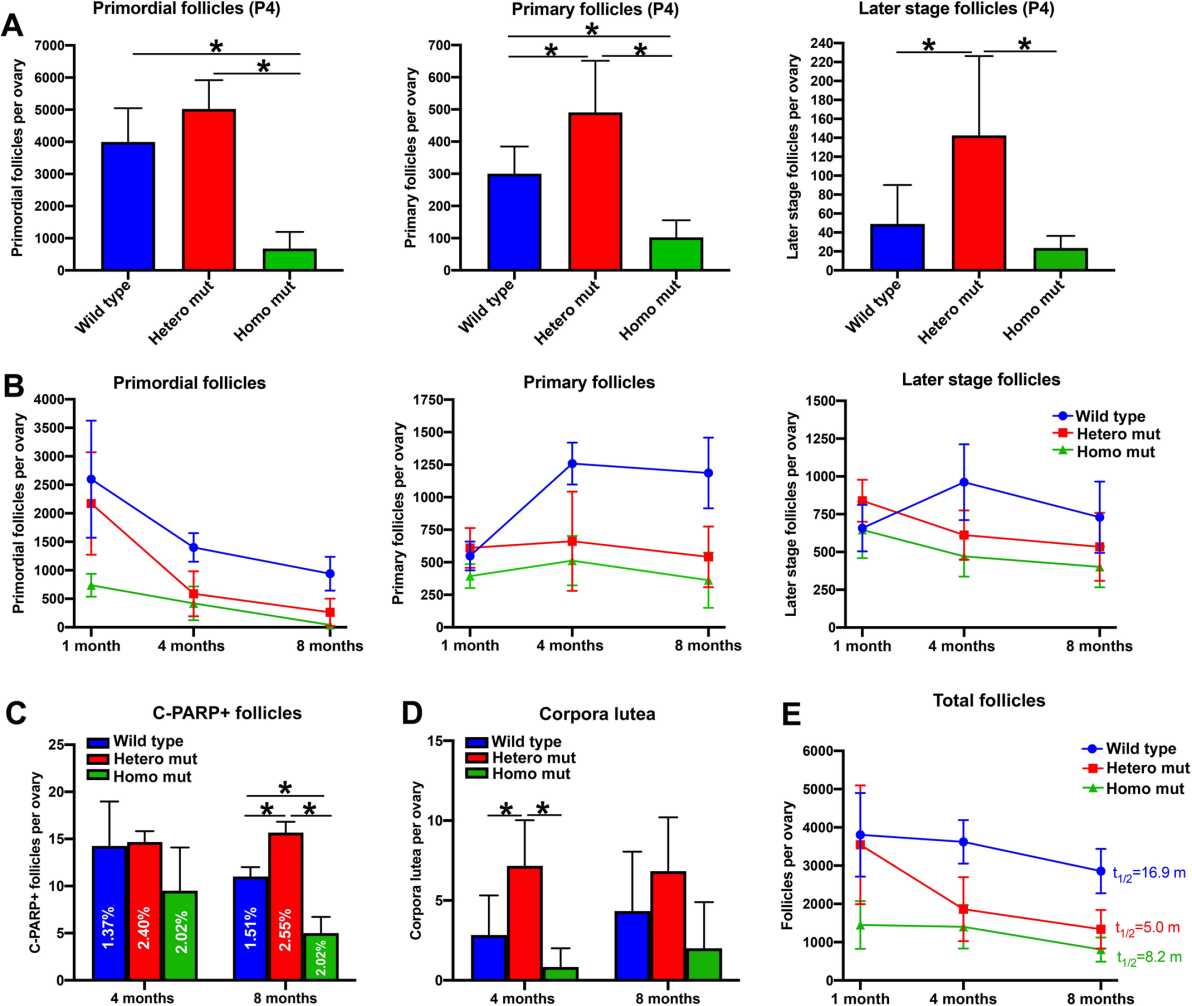
**Dynamics of folliculogenesis in wild-type and *Tex14* mutant ovaries.** (A) Numbers of primordial follicles, primary follicles and later-stage follicles (secondary follicles and beyond) in P4 ovaries. (B) Changes in the numbers of primordial follicles, primary follicles and later stage follicles in adult ovaries from 1 month to 8 months. (C) Numbers of atretic later stage follicle in 4 months and 8 months ovaries. (D) Numbers of corpora lutea in 4 months and 8 months ovaries. (E) Changes in total follicle numbers in adult ovaries from 1 month to 8 months (*n*=6 ovaries for each time point). Error bars present standard deviations. One-way ANOVA was used for statistical analyses, * indicates significant difference with *P* value <0.05; t_1/2_, half-life; m, month.

A previous study found that *Tex14* mutant female mice are fertile and that females of the three genotypes produced roughly the same size litters during the first 6 months (Greenbaum et al., 2009). Here, we observed significant differences in the number of primordial follicles and average primary oocyte size in P4 ovaries of three genotypes (Fig. 4A). These results raised the question of whether differential utilization and/or survival of primordial follicles compensate for their numerical differences.

We first quantified follicles at primordial, primary and later (follicles beyond secondary follicle) stages by using a consistent follicle quantification approach (Pedersen and Peters, 1968; Bristol-Gould et al., 2006; Lei and Spradling, 2013a). At P4, when primordial follicle formation is complete, a homozygous mutant ovary contained significantly fewer follicles at all developmental stages compared with wild type. In contrast, on average, a heterozygous mutant ovary contained 1.3 times more primordial follicles, 1.6 times more primary follicles, and 2.9 times more later stage follicles compared with a wild-type ovary (Fig. 4A). At 1 month of age (sexual maturity), although homozygous mutant ovaries contained only approximately one quarter of the primordial follicles of a wild-type ovary, comparable numbers of primary and later stage follicles were found in wild-type and homozygous mutant ovaries. From 1 month to 4 months, primordial follicles in heterozygous mutant ovaries decline rapidly, which resulted in comparable numbers of primordial, primary and later stage follicles in heterozygous and homozygous mutant ovaries in 4-month ovaries. By 8 months, both heterozygous and homozygous mutant ovaries contained significantly fewer primordial, primary and later stage follicles than wild-type ovaries (Fig. 4B, Fig. S9).

We further examined the number of later-stage atretic follicles in adult ovaries. In 4-month ovaries, although there were no significant differences in the numbers of later-stage atretic follicles between the three genotypes, the percentage of atretic follicles (later stage follicles containing C-PARP+ granulosa cells) was highest in heterozygous mutant ovaries. By 8 months, heterozygous mutant ovaries contained significantly more later stage atretic follicles than did wild-type and homozygous mutant ovaries (Fig. 4C). Quantification on corpora lutea revealed that in the 4-month ovary, on average a heterozygous mutant ovary contained more corpora lutea (7.2±2.9) than those in the wild-type (2.8±2.4) or homozygous mutant (0.8±1.2) ovary. Comparable numbers of corpora lutea were found in the ovaries of all three genotypes by 8 months (Fig. 4D).

From 1 month to 8 months, although heterozygous mutant ovaries started with a comparable number of primordial follicles as wild-type ovaries, the rate of primordial follicle loss was more than 2 times faster. The half-life of the primordial follicle pool from 1 month to 8 months (the time it takes to lose half the amount of primordial follicles) was 4.8 months in the wild type, 2.3 months in heterozygous mutants and 1.7 months in homozygous mutants. Interestingly, compared with heterozygous mutants, total follicles were lost at a slower rate in homozygous mutant ovaries. The half-life of the total follicles from 1 month to 8 months (the time it takes to lose half the amount of total follicles) was 16.9 months in wild-type, 5.0 months in heterozygous mutant and 8.2 months in homozygous mutant ovaries (Fig. 4E). This result indicates that compared with the heterozygous mutants, fewer primordial follicles might be lost by primordial follicle death in homozygous mutants, and thus these primordial follicles were used more efficiently to sustain folliculogenesis.

### TEX14 protein gradually accumulates on intercellular bridges during germline cyst formation

**Fig. 5.**
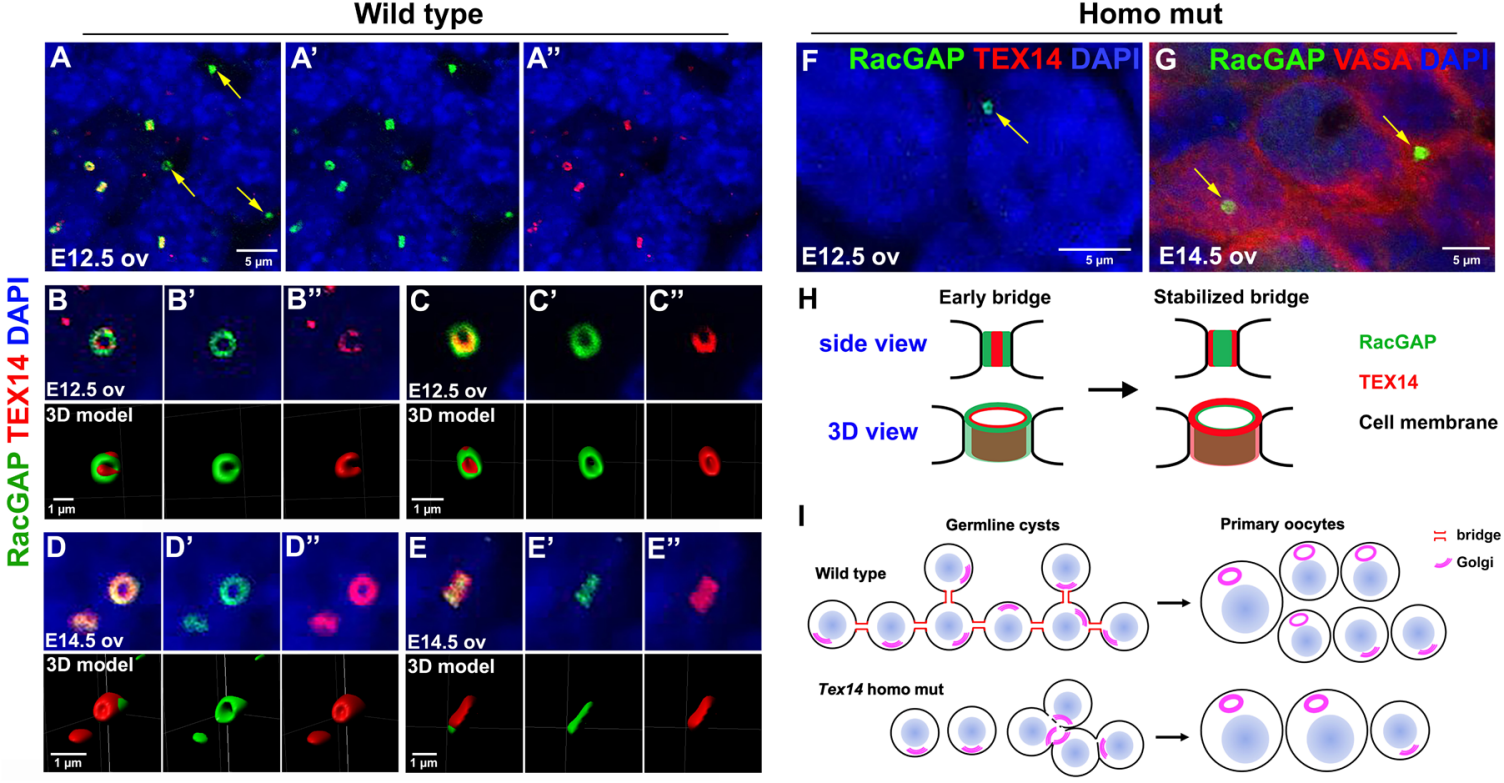
**TEX14 protein gradually accumulates on RacGAP-positive bridges during germline cyst formation.** (A–A″) In E12.5 ovaries, some RacGAP-positive bridges are TEX14 negative (arrows). (B–B″) A cross section of an E12.5 bridge showing TEX14 protein distributed partially on the RacGAP-positive bridge. (C–C″) A lateral section of an E12.5 bridge showing RacGAP protein located at the outer layer of the bridge and TEX14 located at the inner layer of the bridge. (D–D″) A cross section of an E14.5 bridge showing TEX14 protein completely overlapped with RacGAP protein on the bridge. (E–E″) A longitudinal section of an E14.5 bridge showing TEX14 located at the outer layer and RacGAP located at the inner layer of the bridge. *N*=6 ovaries (approximately 200 bridges per ovary) were analyzed at each time point. (F,G) A few RacGAP-positive bridges (arrows) were found in E12.5 and E14.5 *Tex14* homo mutant ovaries. (H) A diagram showing the process of bridge stabilization via TEX14 accumulation on the bridge and the change in its location to the outer layer of the bridge. (I) A diagram demonstrating defects in germ cell connectivity and oocyte differentiation in *Tex14* homo mutant ovaries.

Our previous study revealed that on average, each PGC proliferates to form two-cell or four-cell cysts at E11.5, and four-cell, eight-cell or 16-cell cysts by E12.5. By E14.5 each PGC has produced a 30-cell clone on average, however, cyst fragmentation gradually disrupts synchrony throughout the clone as a whole (Lei and Spradling, 2013b). To further characterize the mechanisms underlying defective intercellular bridge formation and altered germline cyst formation in *Tex14* mutants, we stained E12.5 and E14.5 ovaries with a RacGAP antibody that labels early bridges and a TEX14 antibody that labels stable bridges (Greenbaum et al., 2007). We found that in E12.5 wild-type ovaries, the RacGAP antibody labels significantly more bridges than the TEX14 antibody. All TEX14-positive bridges were RacGAP positive, however, only 51±4% of the RacGAP-positive bridges were TEX14 positive in E12.5 ovaries (Fig. 5A–A″). By E14.5, when germ cell mitosis mostly ceases, 92±5% of RacGAP-positive bridges were TEX14 positive.

We further examined the distribution of RacGAP and TEX14 protein on the early (E12.5) and mature (E14.5) intercellular bridge by 3D modeling of antibody-stained bridges. When we observed bridges from the cross section of the ring in E12.5 ovaries, RacGAP was found to distribute throughout the ring with several spots staining for higher levels of RacGAP protein. TEX14 was not accumulated on the ring completely yet, and appeared as several patches along the RacGAP-positive ring (Fig. 5B–B″). This observation is consistent with the role of RacGAP in cytokinesis, and the function of TEX14 in stabilizing the midbody rings into stable intercellular bridges. When we examined the pattern of RacGAP and TEX14 distribution from the lateral face of the bridges in E12.5 wild-type ovaries, RacGAP distributed on the outer rim of the bridge that associate with the cell membrane, and TEX14 distributed on the inner layer of the RacGAP-positive ring (Fig. 5C–C″). At the cross sections of a bridge in the E14.5 ovary, both RacGAP and TEX14 protein distributed throughout the ring (Fig. 5D–D″). When observing the bridge from the lateral face, RacGAP was exclusively enriched in the inner layer of the ring, while TEX14 protein localized on the outer rim of the ring that interacts directly with the cell membrane (Fig. 5E–E″). This observation suggests that during ring formation, TEX14 replaces RacGAP and interacts with the cell membrane at certain location to stabilize intercellular bridges and maintain cyst structure (Fig. 5H).

In *Tex14* homozygous mutant ovaries, TEX14 protein was not detected by antibody staining, while RacGAP-positive bridges were observed at a decreased frequency (19.8±5.1 germ cells/bridge at E12.5; 18.3±12.8 germ cells/bridge at E14.5) compared with the wild-type germ cells (1.6±0.1 germ cells/bridge at E12.5; 1.9±0.1 germ cells/bridge at E14.5) (Fig. 5F,G). This observation indicates that intercellular bridge formation in fetal ovaries can be initiated but can't be completed without TEX14 protein. Lack of TEX14 did not alter the expression of other cytokinesis proteins (MKLP1, ALIX, CEP55 and TSG101) in E14.5 heterozygous and homozygous mutant ovaries (Fig. S6). The distribution of these proteins on bridges was not identified due to difficulties in visualizing these proteins by immunostaining. These observations suggests that TEX14 may not be essential to block the completion of cytokinesis during germline cyst formation in fetal ovaries, but play a major role in stabilizing the bridges on the cell membrane. This timing of TEX14 accumulation on intercellular bridges is also consistent with the timing of germ cell mitosis defects observed from E12.5 to E14.5 in homozygous mutant ovaries, where cyst formation takes place prior to TEX14-mediated bridge stabilization (Fig. 2I).

## DISCUSSION

Our studies confirm that germline cysts play an important role in mammalian oocyte differentiation despite the fact that *Tex14* homozygous mutant females with disrupted intercellular bridges remain fertile (Greenbaum et al., 2006, 2009). Although TEX14 is essential to complete and stabilize intercellular bridges, its loss does not prevent an initial arrest of cytokinesis and transient assembly of bridge precursors. Before bridges are lost entirely, a significant number of germ cells still become connected via membrane gaps or other structures into cysts that are able to generate oocytes. This explains why *Tex14* mutant germ cells were observed to remain clustered during fetal stages. By studying the effects of *Tex14*-induced cyst alterations in both heterozygous and homozygous mice, we also acquired new insights into the roles cysts play in oocyte differentiation, including how the number of primary oocytes and the cytoplasmic content of each oocyte may be determined.

Unlike germline cysts in *Drosophila*, in which all 16 sister germ cells remain connected during oogenesis (de Cuevas et al., 1997). Mouse cysts undergo fragmentation during and after cyst formation (Lei and Spradling, 2013b). Cyst fragmentation may serve as a means to produce cysts at ‘manageable’ sizes which allows cytoplasmic enrichment of an appropriate number of germ cells to produce oocytes of an ideal size in a defined time window. In *Tex14* heterozygous mutant ovaries, intercellular bridge size becomes much more heterogeneous (Fig. 1L–N). Incomplete and smaller-sized bridges alter cyst fragmentation and influence oocyte differentiation and folliculogenesis. These observations indicate that intercellular bridges may control cyst fragmentation and oocyte selection. In *Tex14* heterozygotes with smaller, potentially abnormal bridges, these processes may fail to operate normally, leading first to incomplete fragmentation, and then to the production of too many oocytes, thereby reducing the potential of each for cytoplasmic growth.

In homozygous mutant ovaries, even more variation was observed in the nature of cellular interconnections. Some formed cellular syncytia, but the ultimate fate of these cell groups remains to be determined. Most germ cells in *Tex14* homozygotes are connected via fragmented membranes without intercellular bridges, a condition likely to disrupt possible bridge function. Homozygous cysts underwent precocious cytoplasmic enrichment and a lower rate of oocyte differentiation. On average, 16.2% of the E14.5 germ cells became primary oocytes and primary oocytes enriched 6.5 folds of cytoplasm (Fig. S7). During the late stage of cytoplasmic transport in wild-type germline cysts, the detachment of the intercellular bridges from the cell membrane also leads to multiple nuclei in a germ cell. However, multinucleated primary oocytes are never found. This observation suggests that a germ cell not selected to become an oocyte is able to transport its cytoplasm and organelles to a neighboring cell while retaining and eventually eliminating its nucleus. Interestingly, when the average fold increase of germ cell volume (P4/E14.5) is multiplied by the average percentage of germ cells that become primary oocytes (P4/E14.5), the products are around 100 in all three genotypes (Fig. S7). This indicates that germ cell loss from E14.5 to P4 is largely due to cytoplasmic transport from non-surviving germ cells into primary oocytes.

Our present study demonstrated the difference in intercellular bridge formation and function between oogenesis and spermatogenesis. In adult testes, TEX14 is the key protein that stops cytokinesis and converts the midbody ring into a stable intercellular bridge (Greenbaum et al., 2007). In the absence of TEX14, male germ cells that complete cytokinesis become individualized. Although the first cycle of spermatogenesis can initiate and progress to meiotic stages, no spermatids and only a reduced number of spermatocytes were observed in 3 weeks homozygous mutant testes due to early meiotic death (Greenbaum et al., 2006). Our observations on the timing and localization of TEX14 protein in fetal ovaries suggests that TEX14 functions to stabilize bridges during mid to late cyst formation (E12.5-E14.5), instead of blocking cytokinesis. During early cyst formation from E10.5 to E12.5, about half of the RacGAP-positive intercellular bridges were TEX14 negative. These TEX14-negative bridges may enable flexibility of bridges at the cell membrane, which allows the formation of branched cysts observed in E14.5 ovaries (Lei and Spradling, 2016). The stabilization of the bridges to the cell membrane may be achieved by changing the location of TEX14 from the inner ring to the outer ring in order to directly interacting with the cell membrane. Similar inner/outer layer of bridges and bridge stabilization have been well characterized in *Drosophila* gametogenesis. In both male and female cysts, mature ring canals consist of an outer rim closely associated with the plasma membrane and an inner rim that appears less dense in electron micrographs (Mahajan-Miklos and Cooley, 1994). *Drosophila* intercellular bridges also experience a maturation process during which bridge composition changes. Shortly after germ cells complete the fourth round of mitosis, phosphotyrosine epitopes accumulate at the site of the contractile ring and transform these arrested cleavage furrows into mature ring canals (Mahowald, 1971; Yue and Spradling, 1992). TEX14 protein contains a kinase domain, although kinase activity has not been observed *in vitro* (Fig. S1). An interesting difference in intercellular bridges between *Drosophila* oogenesis and mouse oogenesis is the phenomenon of bridge expansion. During *Drosophila* oogenesis, as large-scale cytoplasmic transport takes place, the size of the intercellular bridges expands from 1–2 µm initially to ∼10 µm. Mouse bridges do not expand, but detach from the cell membrane and expand cell–cell connectivity to facilitate large-scale cytoplasmic transport. Precocious cytoplasmic enrichment we observed in homozygous mutant ovaries reveals that stable intercellular bridges also play a role in limiting the rate of cytoplasmic transport during mouse oocyte differentiation.

The present study illuminates how cyst formation and fragmentation influence oocyte number and size. We found that within the cysts with intercellular bridges (in both wild-type and heterozygous mutants), cyst size is negatively correlated with oocyte size. In E14.5 wild-type ovaries, on average each cyst contained six cells, and 24.6% of the E14.5 germ cells became primary oocytes by P4. Primary oocytes increased their cell volume 4.0 times from E14.5 to P4. In heterozygous mutant ovaries, cysts were larger in size with 8.5 cells per cyst. The fate of these large cysts contrasts with the expectation that only one cell per cyst can become an oocyte, Instead, heterozygous *Tex14* cysts show a higher frequency of the oocyte formation (36.5%), but these primary oocytes contain less cytoplasm than wild type. This behavior of *Tex14* heterozygous cysts is one of several indications in our studies that intercellular bridges regulate cyst behavior, in addition to allowing materials to move between linked cells.

We observed a surprising difference in the apparent efficiency of primordial follicle usage between the different *Tex14* genotypes. The efficiency of primordial follicle usage is likely determined by multiple factors, including the number of primordial follicles and the rate of primordial follicle recruitment and primordial follicle death (Reddy et al., 2010; Durlinger et al., 2002; Desai and Rajkovic, 2017; Faddy and Gosden, 1995; Da Silva-Buttkus et al., 2009; Schmidt et al., 2004). We found that although a P4 heterozygous mutant ovary contains a similar number of primordial follicles as wild type, the number of large primary oocytes (greater than 20 µm in diameter) is much lower than that in wild type (Fig. S10). In contrast, a P4 homozygous mutant ovary contains only one fifth the primordial follicles of wild type (Fig. 4A). However, a greater proportion of primary oocytes of large size were observed in the homozygous mutant (Fig. S10). In adult ovaries, heterozygous mutant primordial follicles turned over faster than in wild type, while homozygous mutant follicles showed increased stability. The rapid turnover of heterozygotes could reflect a defect in oocyte contents related to their abnormal selection and inadequate growth. However, the reason for the apparently greater efficiency in follicle usage of *Tex14* homozygotes may be interesting to characterize further. A similar phenotype of efficient folliculogenesis observed in *Tex14* homozygous mutant ovaries was also reported in CCS1 *Sohlh1* mutant mice, in which *Sohlh1* (germ cells express spermatogenesis and oogenesis bHLH transcription factor1) is expressed in germ cells of the *Sohlh*-null mutant mice after E15.5. Despite a significantly reduced number of primordial follicles, the mutant females had normal folliculogenesis and fertility up to 10 months old. Although the size of primary oocytes was not analyzed in the CCS1 *Sohlh1* mutant mice, the reduced size of ovarian reserve and efficient follicle usage observed in this mutant mouse model is consistent with that in *Tex14* homozygous mutant mice (Fig. 4A,E).

In summary, by characterizing the phenotypes of germ cell connectivity, germline cyst fragmentation, oocyte differentiation and folliculogenesis in *Tex14* mutant mouse ovaries, we found additional evidence supporting the importance of development within cysts to oogenesis. In particular, the transfer of cytoplasm between cells within cysts and its accumulation within primary oocytes is a centrally important aspect of mammalian oogenesis. Cytoplasmic transfer did not require normal intercellular bridges in *Tex14* homozygous mutant ovaries, but took place with near wild type efficiency even when cyst structure was severely perturbed. Our results suggest that intercellular bridges contribute to the regulation of cyst fragmentation and oocyte selection, these processes further influence the size of ovarian reserve and oocyte usage in the adult ovary.

## MATERIALS AND METHODS

### Mice

CAG-creER (004682) and R26R-YFP (006148) mouse strains were acquired from the Jackson Laboratory. *Tex14* mutant mouse line was generously provided by Dr. Martin Matzuk's lab at Baylor College of Medicine (Greenbaum, 2006). All mice were maintained at C57BL/6 background and housed and bred according to the protocol approved by the Institutional Animal Care and Use Committee (IACUC) at the University of Michigan (PRO00008693) or the Buck Institute for Research on Aging (A10207). Mice euthanasia in this study was performed by strictly following the protocol from the IACUC. The carbon dioxide (CO_2_) inhalation system in the vivarium facility was used for euthanasia. During euthanasia, the mice were placed into a chamber, in which CO_2_ was added slowly to increase its concentration. The personnel performing euthanasia were required to monitor the process, and wait for at least one minute after no movement, visible inhaling or heartbeat were detected. An approved secondary method was used to ensure the death of the mice.

### Single-cell lineage labelling

Single-cell lineage tracing was carried out by using the protocol published in our previous study (Lei and Spradling, 2013b; Lei and Spradling, 2016). Briefly, a single dose (0.4 mg per 40 g body weight) of tamoxifen was injected into female (*R26R^YFP/YFP^;Tex14^+/−^*) mice that were plugged by male (*CAG-creER^+/−^; R26R^YFP/YFP^;Tex14^+/−^*) mice at E10.5. Fetal ovaries and testes were collected at E14.5 and lineage-labeled clones were revealed by YFP antibody staining.

### Whole-mount immunostaining and fetal germ cell quantification

Fetal ovaries and testes were dissected in cold phosphate buffer saline (PBS) and fixed in 4% paraformaldehyde (PFA) for 1 h at 4°C. Tissues were washed in PBST_2_ (PBS with 0.1% Tween 20 and 0.5% Triton X-100) and incubated in primary antibodies (Table S1) at 4°C overnight. The next day, tissues were washed in PBST_2_ and incubated in secondary antibodies at 4°C overnight. The tissues were washed in PBST_2_ and incubated in DAPI at room temperature for 30 mins to stain nuclei. The stained gonads were mounted on glass microscope slides using imaging spacers and analyzed using confocal microscopy.

Fetal gonads stained with anti-VASA antibody were used for germ cell number quantification. Germ cells of the largest cross section were imaged using confocal microscopy and counted using Image-J image analysis software. The germ cell diameter was obtained by averaging two diameters at the largest cross section of the cell using Image-J. The thickness of the ovary was obtained by measuring the distance from the top to the bottom of the tissue. The total number of germ cells per gonad was determined by using the following calculation: gonad thickness/germ cell diameter×germ cell number on the largest cross section. Six ovaries from individual mice of each genotype were used for germ cell quantification.

### Germ cell diameter measurement and B-body quantification

Fetal/neonatal ovaries stained with GM130 and VASA antibodies were imaged using confocal microscopy at the Z=1 µm per scan. To measure germ cell diameter, an area of 100 µm wide and 100 µm deep to the ovarian surface were chosen in each ovary. For E12.5 and P0 ovaries, all germ cells in the area were analyzed. For P4 ovaries, oocytes in the developing primary follicles surrounded by cuboidal granulosa cells were not included in diameter measurement. Two vertical diameters of the biggest cross section of each germ cells were measured, and the average value (R_ave gc_) was used to represent the diameter of the germ cell. The volume of the germ cells (V_gc_) was calculated using the equation: V_gc_=4/3×3.14×R_ave gc_^3. The B-body is defined as a circular Golgi complexes in VASA positive germ cells. Two optical sections with 10 µm interval were chosen for quantifying the number of total germ cells and the number of germ cells containing a B-body.

### Intercellular bridge quantification, diameter measurement and 3D modeling

Fetal ovaries stained with RacGAP, TEX14 or VASA antibodies were imaged using confocal microscopy at the Z=0.5 µm per scan. Three optical sections with 10 µm interval were chosen for quantifying the number of RacGAP-positive bridges, the number of TEX14-positive bridges and the number of germ cells at the section using Image-J. The diameters of the TEX14-positive bridges were measured using the measure tool of Image J software. For each bridge, two vertical diameters at the biggest cross section of the bridge were measured, and the average value was used to represent the diameter of the bridge. For 3D bridge modeling, IMRIS software was used to view and generate three-dimensional modeled images of the bridges.

### Follicle quantification of postnatal and adult ovaries

Postnatal day 4 and adult ovaries were fixed in 4% PFA overnight at 4°C. After fixation, ovaries were washed in PBS and incubated in 30% sucrose overnight before embedding in optimal cutting temperature (OCT) compound. Serial sections were cut at 10 µm of the entire ovary. Sections were stained by using VASA antibody to reveal oocytes. Follicles were counted on every fifth section. The number of follicles per ovary were calculated as follicles/section×total sections. After follicle quantification, sections were stained by using Hematoxylin and Eosin for corpus luteum (CL) quantification. Based on the average size of CL (400 µm), CL were counted at every 40 sections and were summed for the total number of CL in the ovary (Lei et al., 2010; Numazawa and Kawashima, 1982). Six ovaries from individual mice of each genotype were used for follicle quantification at each time point. The half-life (t_1/2_) of follicles was calculated as: t_1/2_=(final age - initial age)/log1/2(final follicle number/initial follicle number).

### Apoptotic and mitotic germ cell assay

Apoptosis assay: fetal ovaries were fixed and stained by using the cleaved-PARP antibody. The number of cleaved-PARP-positive cells in an ovary was quantified by counting through serial confocal sections of the ovary using Image-J. Percentage of apoptotic cells=% (cleaved- PARP^+^ cells/total germ cells). Three ovaries of each genotype were analyzed.

Proliferation assay: fetal ovaries were dissected and incubated with EdU (50 µM) for 30 mins in DMEM/F12 medium with 3 mg/ml bovine serum albumin. The ovaries were fixed and stained with EdU labeling kit and VASA antibody. EdU^+^ and VASA^+^ cells in a 100 µm cubical area of the serial confocal images at the periphery region of the ovary were quantified using Image-J. Percentage of mitotic germ cells=% (EdU^+^; VASA^+^ cells/ VASA^+^ cells). Six ovaries from individual mice of each genotype were analyzed.

### Electron microscopy

Electron microscopic imaging was done by Mike Sepanski at the Carnegie Institution as previously described (Yue and Spradling, 1992) and the Microscopy and Image Analysis Laboratory (MIL) at the University of Michigan. Six ovaries from individual mice of each genotype were analyzed.

### Western blotting

Protein was exacted from E14.5 fetal ovaries and testes using RIPA lysis buffer. 15 mg protein sample was run using the NuPAGE Bis-Tris gel and transferred onto a PVDF membrane. The membrane was washed with TBST (tris-buffer saline, 0.1% Tween 20) and blocked with 5% bovine serum albumin (BSA) at room temperature for 1 h. The membrane was incubated with primary antibodies diluted in TBST (Table S1) at 4°C overnight. After washing the membrane with TBST, the membrane was incubated with an HRP-conjugated secondary antibody diluted in TBST at 4°C overnight. The membrane was developed using the ECL western blotting substrate. *N*=40 gonads of each genotype were pooled for each western blot sample. *N*=3 experiments were conducted.

### Statistics

All data was presented as mean±s.d. Non parametric *t*-tests were run to analyze the difference between two experimental groups. Multiple experimental groups were analyzed using one-way ANOVA. *P* value level of at least *P*<0.05 was considered to be statistically significant.

## Supplementary Material

Supplementary information
