# Supplementary material for: Altered germline cyst formation and oogenesis in Tex14 mutant mice
Source: Biol Open. 2021 Jun 22;10(6):bio058807. doi: 10.1242/bio.058807 (PMC8249907; doi:10.1242/bio.058807)
Supplement: Supplementary information [file biolopen-10-058807-s1.pdf]

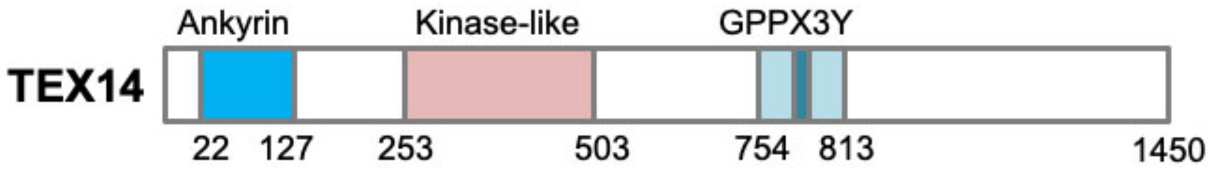

Fig S1. Motifs of the mouse TEX14 protein. (Adapted from Iwamori T et al., Molecular and Cellular Biology, 2010; 30(9):2280-92).

Biology Open • Supplementary information

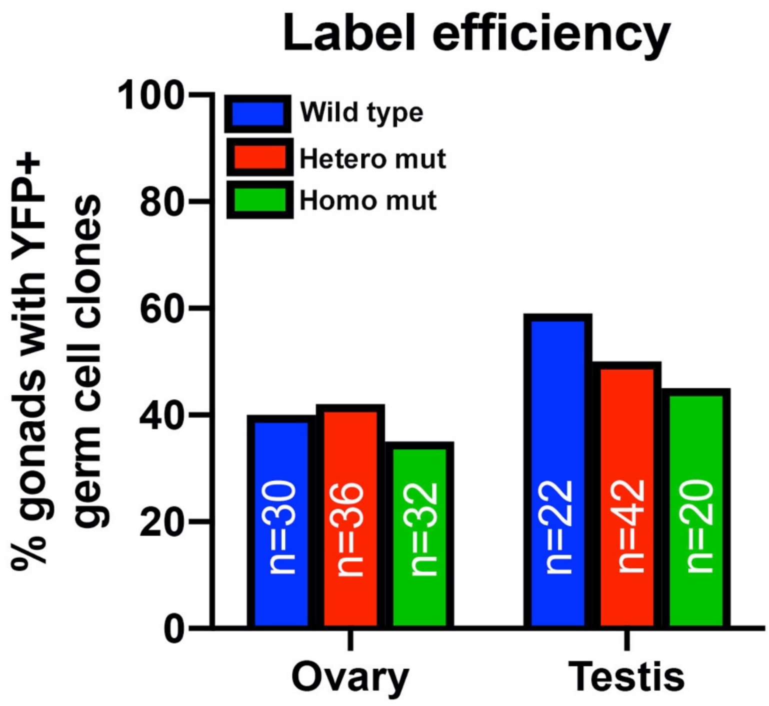

Fig S2. Single-PGC lineage labeling efficiency. The efficiency of single PGC-lineage labeling shown by the percentage of fetal gonads containing YFP positive germ cell clones in wild type and *Tex14* mutant mouse fetal gonads.

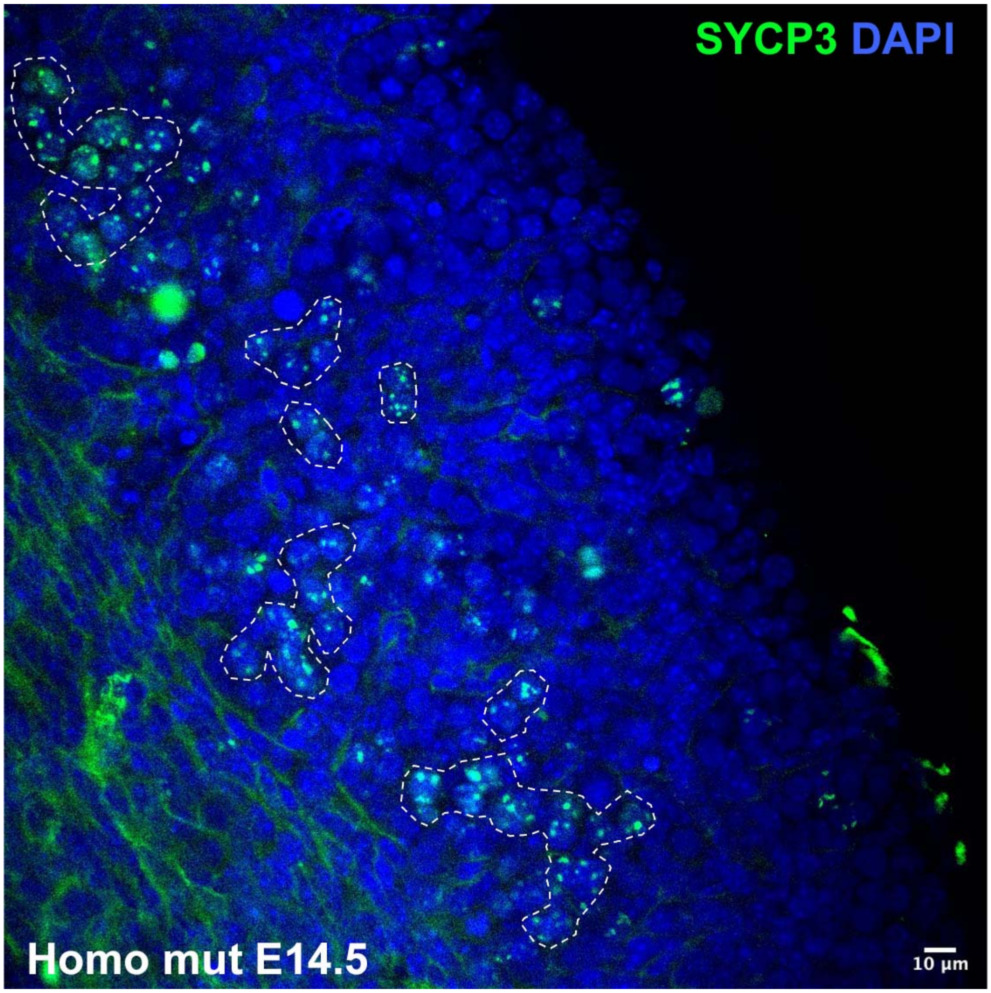

Fig S3. Clustered SYCP3-positive cells observed in the E14.5 homozygous mutant ovary. (n=6 ovaries).

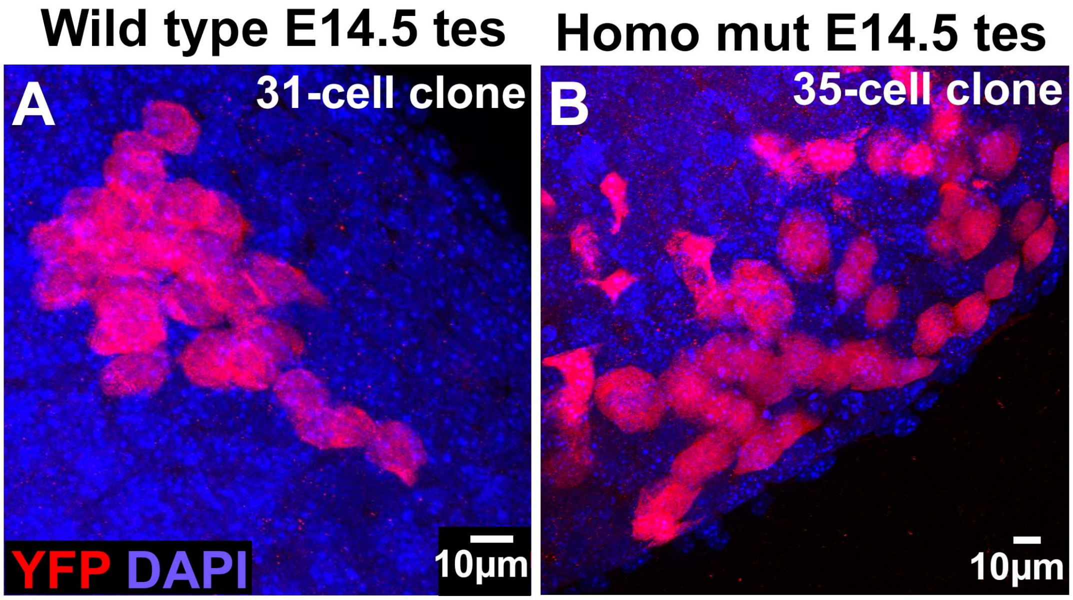

Fig S4. The germ cell clone revealed by single-PGC lineage tracing in E14.5 testes. (A) A YFP-positive germ cell clone containing a germline cyst in the E14.5 wild type testis (n=22 testes). (B) A YFP-positive germ cell clone containing many smaller cysts in the E14.5 homozygous mutant testis (n=20 testes).

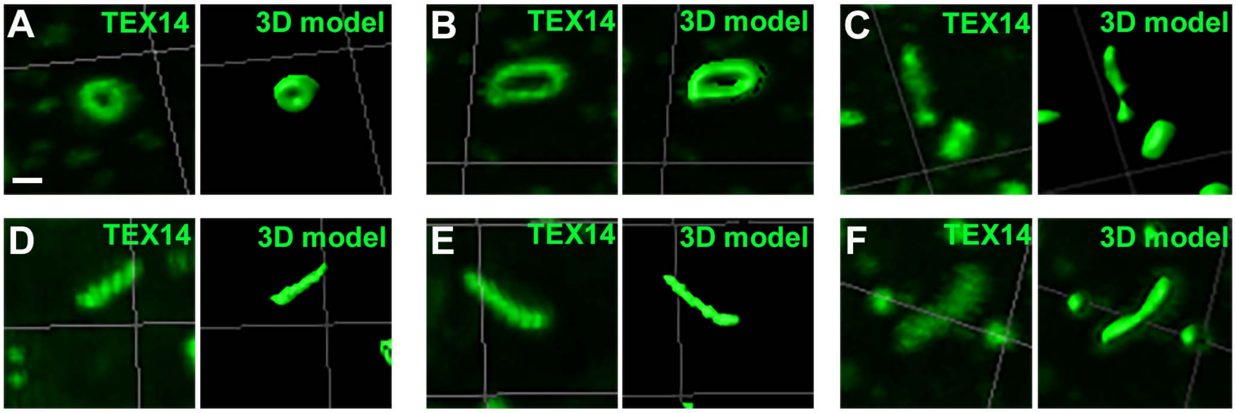

Fig S5. 3D images of TEX14 antibody-stained intercellular bridges and the models of these bridges observed in E14.5 *Tex14* heterozygous mutant ovaries. (A, B) Ring-shaped intercellular bridges. (C-F) Fragmented intercellular bridges. (N=6 ovaries were examined).

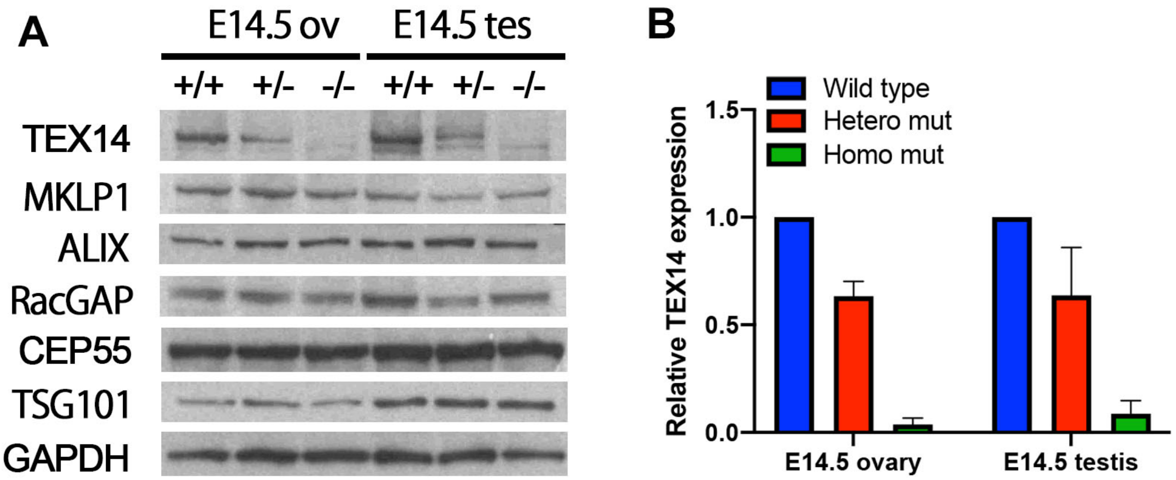

Fig S6. The expression of cytokinesis proteins. (A) The expression of TEX14, MKLP1, ALIX, RacGAP, CEP55 and TSG101 in E14.5 wild type (+/+), heterozygous mutant (+/-) and homozygous mutant (-/-) gonads examined by western blot. N=40 gonads of each genotype were pooled for each western blot sample. N=3 experiments were conducted. (B) The relative protein level of TEX14 in E14.5 wild type and mutant gonads. Error bars present standard deviations

Biology Open • Supplementary information

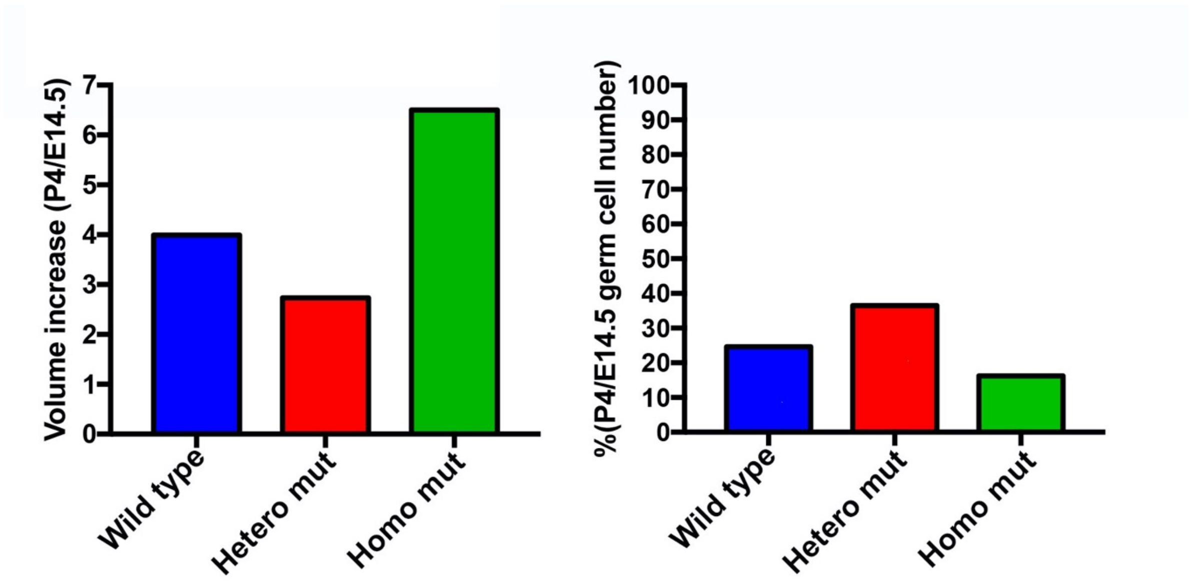

Fig S7. The fold of germ cell volume increase from E14.5 to P4 and the percentage of E14.5 germ cells differentiate into primary oocytes. N=6 ovaries (approximately 100 germ cells/ovary) at each time point were used for germ cell volume analyses. Total number of the germ cells per ovary at each time point were used for germ cell number analyses.

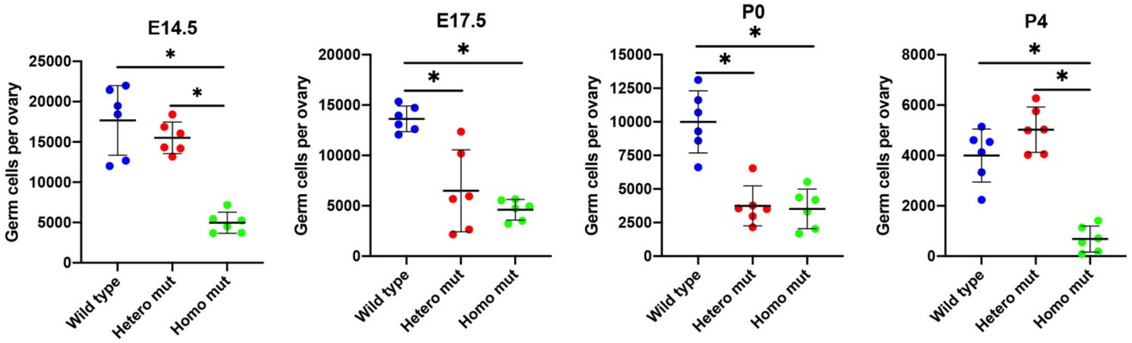

Fig S8. Number of germ cells in the fetal ovary during oocyte differentiation. N=6 ovaries at each time point were used for analyses. One-way ANOVA was used for statistical analyses, * indicates significant difference with P value <0.05. Error bars present standard deviations.

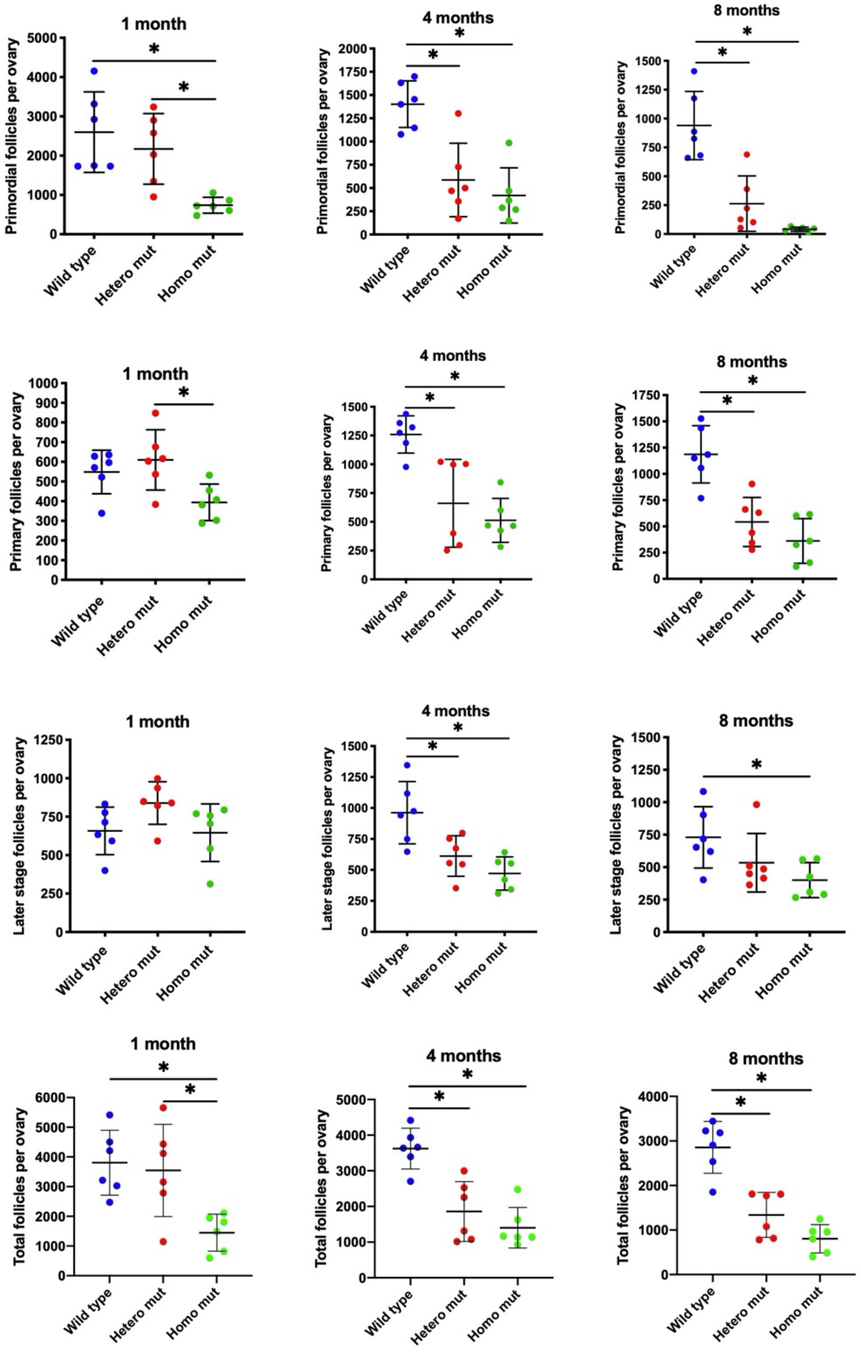

Fig S9. Number of follicles in the adult ovary from 1 month to 8 months. N=6 ovaries at each time point were used for analyses. One-way ANOVA was used for statistical analyses, * indicates significant difference with P value <0.05. Error bars present standard deviations.

Biology Open • Supplementary information

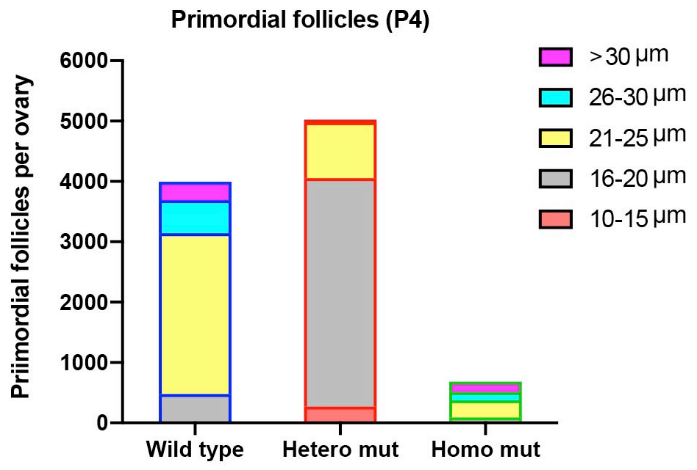

Fig S10. Proportion of primary oocytes based on cell diameter (prop $_{\text{oocyte size}}$) in the ovarian reserve of P4 ovaries. The percentage of primary oocytes at each size range (percentage $_{\text{oocyte size}}$) was calculated based on germ cell diameter measurements in P4 ovaries (Fig 3H). The prop $_{\text{oocyte size}}$ = average number of primordial follicles per ovary x percentage $_{\text{oocyte size}}$ at each size range.

Biology Open • Supplementary information

Table S1. Antibodies used in the manuscript.

Antibodies	Vendors and antibody validation profile	Dilution
TEX14	Protein Tech (18351-1-AP; Lot# was not provided by the vendor) https://www.ptglab.com/products/TEX14-Antibody-18351-1-AP.htm	1:200 (IF) 1:1000 (WB)
RacGAP1 (A-6)	Santa Cruz Biotechnology (sc 271110; Lot# F0612) https://www.scbt.com/p/rac-gap1-antibody-a-6?requestFrom=search	1:100
GM130	BD Biosciences (610822; Lot# 7163678) https://www.bdbiosciences.com/us/reagents/research/antibodies-buffers/cell-biology-reagents/cell-biology-antibodies/purified-mouse-anti-gm130-35gm130/p/610822	1:100
MVH	Abcam (ab13840; Lot# GR3218256) https://www.abcam.com/ddx4--mvh-antibody-ab13840.html	1:200
Sodium Potassium ATPase	Abcam (ab76020; Lot# GR3184452-3) https://www.abcam.com/sodium-potassium-atpase-antibody-ep1845y-plasma-membrane-loading-control-ab76020.html	1:200
TRA98	B-Bridge (73-003; Lot# 170819B) https://www.asone-int.com/wp-content/uploads/2017/05/73-003_73-003_Datasheet.pdf	1:500
Cleaved Parp (Asp214)	Cell Signaling (94885S; Lot# 1) https://www.cellsignal.com/products/primary-antibodies/cleaved-parp-asp214-d6x6x-rabbit-mab-rodent-specific/94885?Ntk=Products&Ntt=94885	1:200
GFP	Aves Labs (GFP-1020; Lot# GFP697986) https://www.aveslabs.com/products/green-fluorescent-protein-gfp-antibody?_pos=1&_sid=8140431bd&_ss=r	1:1000
EdU (labeling kit)	Invitrogen (C10337; Lot# 1741650) https://www.thermofisher.com/order/catalog/product/C10337#/C10337	1:200 for EdU incubation
SCP3	Abcam (ab15092; LotT# 823899) LEI, L. & SPRADLING, A. C. 2013b. Mouse primordial germ cells produce cysts that partially fragment prior to meiosis. *Development,* 140**,** 2075-81.	1:200
MKLP1	Santa Cruz Biotechnology (sc-867; Lot# B2310) https://www.scbt.com/p/mklp-1-antibody-n-19?requestFrom=search	1:500
ALIX	BioLegend (634502; Lot# was not provided by the vendor) https://www.biolegend.com/en-us/products/purified-anti-alix-antibody-4469	1:500

Biology Open • Supplementary information

RacGAP1	Protein Tech (13739-1-AP; Lot# was not provided by the vendor) https://www.ptglab.com/products/RACGAP1-Antibody-13739-1-AP.htm	1:500
CEP55	Abcam (ab170414; Lot# GR128676) https://www.abcam.com/cep55-antibody-epr11944b-ab170414.html	1:500
TSG101	Santa Cruz Biotechnology (sc-7964; Lot# H1319) https://www.scbt.com/p/tsg-101-antibody-c-2?requestFrom=search	1:500
GAPDH	Cell Signaling (5174; Lot# 7) https://www.cellsignal.com/products/primary-antibodies/gapdh-d16h11-xp-rabbit-mab/5174?Ntk=Products&site-search-type=Products&N=4294956287&Ntt=gapdh&fromPage=plp	1:10000

Biology Open • Supplementary information
